# Prevention of Decay of the Teeth

**Published:** 1880-08

**Authors:** R. Russell


### PREVENTION OF DECAY OF THE TEETH.

*Read before the Tennessee State Dental Society.*

BY DR. R. RUSSELL.

This is a subject that has received very little attention from the dental profession and yet it is one of the greatest importance We are reminded every day that the teeth do decay, and that at a very rapid rate, and as the enamel has no vital power to resist decay, it must sooner or later be destroyed unless some means can be employed for its arrest. I propose to give my views and experience which will embrace a period of over thirty years of active practice, twenty-six of which have been spent in middle Tennessee, where good dental operations are as highly appreciated as in any part or the Union, owing to the fact that skillful and honest dentists were located here at a very early period, their operations proved so satisfactory that much is expected of the dental surgeon at the present day, and I am grieved to say that I believe that there has been no advance in our profession so far as saving the teeth is concerned since the days of Badger and Wheeler, who were both great believers in separating the teeth, which I shall endeavor to show is the only true way in which the teeth can be preserved for any great length of time. Without going into any elaborate description as to the cause of caries, whether it be on account of the acidity of the saliva or lodgement of particles of food between the teeth, or hereditary influence, it is self evident that in ninety-nine persons out of every hundred there are carious teeth, and it requires a vast army of dentists in the Union as well as Great Britian to try to keep them in order. It is estimated that there are in America at least 12,000 dentists, and that the annual consumption of pure gold made into foil for this country alone will amount to three tons, costing over $3,000,000, there are also various other articles too numerous to mention which are used for filling teeth. Besides gold, 3,000,000 artificial teeth are manufactured every year to supply those that are lost to the American people. It is estimated that 20,000,000 teeth are extracted every year in America, a great majority of which has received dental operations by plugging. With 12,000 dentists, 3 tons of gold, besides all the cheaper materials which are in constant use, the American people lose from carious teeth 20,000,000 every year, it would appear from the foregoing facts that there certainly must be something wrong somewhere, and that the average dentist falls far short of what his patrons ought to expect of him.

How shall caries of the teeth be prevented? Some dentists hold that with proper care in passing floss silk between the teeth and great hygienic treatment in keeping them clean, that the caries in a great measure can be prevented, but this falls far short of the mark. Imperfect enamel can in many instances be observed upon teeth as soon as they are erupted, especially is this the case with sixth-year molars, the imperfections to which I refer are upon their crowns and unless they are arrested by plugging or filing they will soon be destroyed, all the crown cavities in all the teeth must be attended to in the same manner as soon as disease makes its appearance. There is no known way by which caries in a majority of cases can be prevented in the crowns of the teeth, and that the enamel is not a preserver of the teeth is proven to the dentist every day he is in practice, and the sooner he gives up this most absured notion the better it will be for the community at large. So far we have had very little to say in regard to the prevention of caries in the teeth, but we now come to the subject with a full and practical knowledge that caries of the teeth can in a great majority of cases be prevented in the approximal surfaces.

The central incisors of the permanent set make their appearance from 6 to 8 years; lateral, 7 to 9 years; canines, 12 to 13 years; first bicuspid, 9 to 10 years; second bicuspid, 11 to 12 years; first molar, 6 to 7 years; second molar, 12 to 13 years; wisdom tooth from 17 to 21 years. It will be observed that it is about six years from the time the incisors are cut before the set of teeth are complete, save the wisdom teeth. Now, if parents or guardians will present their children to the dentist as soon as the incisors make their appearance, he should be held responsible should they decay, so as to require plugging through life; the approximal surfaces should be examined with the greatest care, and should there be the slightest appearance of decay, it should be polished off, the teeth should be separated with the tape, first applying one thickness and in a few hours another, which will separate them sufficiently for all practical purposes, it is frequently the case that it can be polished off with tape and pumice stone followed with buckskin and rouge, which will give it a smooth and polished surface and prevent its again making its appearance, provided the teeth are hard and the little patient keeps them thoroughly cleansed, but should the teeth even at this early age, have so far decayed that the foregoing method should prove ineffectual, we must resort to more heroic means for the prevention of the disease.

Next comes the file, thanks to Stubs and Froid, long have I used your files and thousands of teeth would have been forever lost without your most valuable and useful instruments: but all thanks to Arthur for his most beautiful and charming little disks, which will perform the same amount of work in one-fourth of the time. We must remember that we are treating incipient caries, for the prevention of further decay, and this in every case can be successfnlly done if the proper separation is made and made so that the teeth can never come together again. The separation should be made something in the shape of a new moon, great care being taken not to disfigure the labial surfaces of the teeth and there is no necessity for that, as the moon shaped separation is on the lingual surface and seldom need even to extend to the labial surface, the lateral incisors, whenever caries has extended into the dentine should be treated in the same manner, and if proper care is afterwards taken, they will in a great majority of cases save the teeth from further decay and consequently they will have no need of gold plugs. The majority of people as well as dentists have a great horror of the file, they fear lest the enamel will be broken, as they regard it as the protector of human teeth, there was never a greater fallacy, the dentine has more vitality and is a much better protector than the enamel, it has more recuperative power when diseased, it sometimes take on healthy action; enamel has no power to resist disease when once attacked, it goes on until the tooth is completely destroyed.

Let us frown down this foolish idea, teach the people that the disk and file are our most useful instruments. As age advances and the bicuspids, molars and canine teeth make their appearance, let us separate and polish them in a manner so that they can not approximate, and we can do away with nine-tenths of the approximate fillings, saving ourselves great annoyance and our patients a great amount of suffering. To accomplish this result it is necessary that the little children and their parents should be instructed to visit the dentist at an early age, parents must be taught this and who have we to teach them? they seldom go to the dentist with their children at this early age, the family physician knows nothing about the teeth, and thus they are left to grope in the dark until the incisors are decayed; molars, bicuspids and all the teeth are a complete wreck, in many cases before the dentist is called upon.

To obviate this terrible want of knowledge, the doctor and dentist should be educated in the same school, taught by the same professors. The doctor is with the mother when the child comes into the world, he should be its guardian angel, he watches over him in his hours of pain and anguish, when he is cutting his teeth, his solicitude for his welfare is great, but should he be suffering with pain from the exposure of a nerve or pulpitis or periostitis, the doctor is powerless to give the proper advice, but you educate the M. D. and D. D. S. in the same school, teach the M. D. the great importance of the dental art, enlighten him in relation to the great importance of the human teeth, and you have in the M. D. a friend and counsellor that will be of great value to suffering humanity as well as to dentist and doctor.

